# Printing of small molecular medicines from the vapor phase

**DOI:** 10.1038/s41467-017-00763-6

**Published:** 2017-09-27

**Authors:** Olga Shalev, Shreya Raghavan, J. Maxwell Mazzara, Nancy Senabulya, Patrick D. Sinko, Elyse Fleck, Christopher Rockwell, Nicholas Simopoulos, Christina M. Jones, Anna Schwendeman, Geeta Mehta, Roy Clarke, Gregory E. Amidon, Max Shtein

**Affiliations:** 1 Materials Science and Engineering, University of Michigan, Ann Arbor, 48109 USA; 2 College of Pharmacy, University of Michigan, Ann Arbor, 48109-1065 USA; 3 Department of Physics, University of Michigan, Ann Arbor, 48109-1040 USA; 4 Chemical Engineering, University of Michigan, Ann Arbor, 48109-2136 USA; 5 Biomedical Engineering, University of Michigan, Ann Arbor, 48109-2099 USA

## Abstract

There is growing need to develop efficient methods for early-stage drug discovery, continuous manufacturing of drug delivery vehicles, and ultra-precise dosing of high potency drugs. Here we demonstrate the use of solvent-free organic vapor jet printing to deposit nanostructured films of small molecular pharmaceutical ingredients, including caffeine, paracetamol, ibuprofen, tamoxifen, BAY 11-7082 and fluorescein, with accuracy on the scale of micrograms per square centimeter, onto glass, Tegaderm, Listerine tabs, and stainless steel microneedles. The printed films exhibit similar crystallographic order and chemistry as the original powders; controlled, order-of-magnitude enhancements of dissolution rate are observed relative to powder-form particles. In vitro treatment of breast and ovarian cancer cell cultures in aqueous media by tamoxifen and BAY 11-7082 films shows similar behavior to drugs pre-dissolved in dimethyl sulfoxide. The demonstrated precise printing of medicines as films, without the use of solvents, can accelerate drug screening and enable continuous manufacturing, while enhancing dosage accuracy.

## Abstract (web summary)

Traditional approaches used in the pharmaceutical industry are not precise or versatile enough for customized medicine formulation and manufacture. Here the authors produce a method to form coatings, with accurate dosages, as well as a means of closely controlling dissolution kinetics.

## Introduction

Much of drug research and development now focuses on high potency and personalized medicines, and on alternative delivery vehicles^1–3^ (e.g., dermal and buccal patches, biodegradable implants, etc.). However, traditional approaches are not precise or versatile enough for customized medicine formulation and manufacture^4^. Severe trade-offs exist between drug discovery rate, dose customizability, and manufacturing scalability. Here we describe an approach that produces coatings with accurate, customizable dosages, as well as a means of closely controlling dissolution kinetics of active pharmaceutical ingredients (APIs). As proof-of-principle, we demonstrate the printing of widely used yet poorly soluble cancer drugs (BCS II type)^5^, among other APIs, achieving enhanced bioavailability. The combination of improved process control for dose accuracy and consistency, scalable yet customizable process, and controlled dissolution kinetics suggests a pathway for accelerating drug discovery and manufacture.

There are several challenges on the road from drug discovery, to formulation, to screening, and to manufacturing. For example, low solubility and dissolution rates pose severe limitations for new drug discovery^6, 7^. Micronization and nanonization^8^ techniques have been developed to enhance the bioavailability by accelerating the dissolution of an API, commonly made in powder form to increase the surface area to volume ratio^9^ and, hence, dissolution rate. However, mechanical methods (e.g., powder milling, high-pressure homogenization) for producing small particles are energy- and time-consuming, while the resulting nanoparticles may lack stability during storage and controlled release^8^. Formulating medicines with nanoparticles is also challenging, since homogeneity and stability are difficult to achieve due to particle agglomeration and changes in crystallinity^8^, compromising batch-to-batch consistency and safety, particularly for high-potency APIs (HPAPIs). Furthermore, prior to approval for general use, drugs undergoing initial testing are typically dissolved in organic solvents, dimethyl sulfoxide (DMSO) being one of the most common, leading to erroneous estimation of drug efficacy and bioavailability, as compared to direct application of the API. The lack of rapid polymorph phase screening methods combined with limited drug amounts leads to higher candidate attrition rates during drug discovery^10^. Finally, traditional process requirements to achieve high-throughput manufacturing compete with those required for personalization of dosage^11, 12^. In contrast, film-form drug delivery vehicles have been demonstrated^13, 14^ and have the potential to enable high throughput, continuous manufacturing, and personalized dosage simultaneously. For topical applications, thin film pharmaceutical coatings are used in transdermal drug delivery systems, such as patches and microneedles^15^. For localized drug delivery, thin films of APIs are applied in the form of coatings onto various delivery vehicles (wafers, rods)^1^. Film-form drug systems are also used for transmucosal and oral drug delivery, providing the means for rapid drug dissolution and transport to the systemic circulation^16, 17^. Manufacturing of film-form drugs includes dispersion of API particles in a polymer matrix by mixing, dipping, or spraying, followed by polymer casting or extrusion^18^. These approaches, however, suffer from limited particle dispersion, stability, and drug loading, especially when working with nanoparticles^14^.

**Fig. 1:**
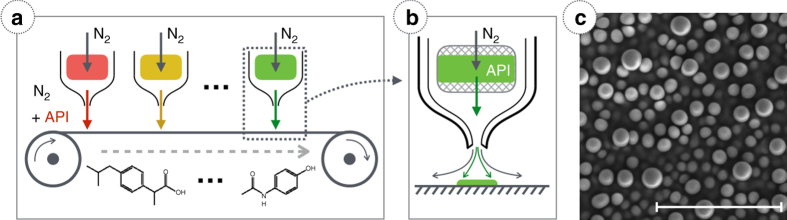
OVJP system schematic. **a**, **b**—OVJP operation principle. Inert carrier gas passes through an evaporation zone picking up the organic vapor and jetting onto a cooled substrate where the organic molecules selectively condense. **c** Fluorescein nanolobes grown by OVJP. *Scale bar* 5 μm

More recently, vacuum thermal evaporation (VTE) was used to create micro- and nano-structured thin films of drugs^19^. In VTE, the source material is heated in vacuum, where the molecular mean free path is long, resulting in line-of-sight deposition by physisorption on a cooled surface^20^. Drug films obtained by VTE can exhibit enhanced dissolution due to increased surface area, while high dosing accuracy is in principle possible. Although VTE is a widely used technique for inorganic and organic material deposition, its scalability for the pharmaceutical industry is limited due to relatively low, controlled deposition rates (on the order of Å per s) and low material yield. Additionally, due to the deposition mechanism, some morphologies are difficult to access^21–23^, while significant cross-contamination risk of the common evaporation chamber and parasitic deposits makes it poorly suited for drug discovery. To overcome the challenges outlined above, we adapt a process originally developed to achieve continuous, solvent-free, large-scale, high-throughput, yet ultra-precise printing of small-molecular organic semiconductors: organic vapor jet printing (OVJP)^22^. It proceeds by thermally evaporating the substance (here, an API) into a stream of inert carrier gas (e.g., nitrogen), followed by jetting onto a substrate, where the API forms a film (Fig. 1a, b). Figure 1b demonstrates the OVJP working principle. The organic material is evaporated into a carrier gas; the mixture of evaporated material and carrier gas is jetted onto the cooled substrate, where the organic material condenses. The process is controlled via several parameters^20^, which regulate the deposition rate, the deposit shape, as well as the resulting film morphology. Previously^21–23^, we demonstrated how OVJP can provide morphological control and generate thick (>500 nm) and large area deposits, while also enabling additive patterning with high material utilization efficiency (>70%). When film thickness exceeds 200 nm, the films can evolve into unique three-dimentional nano and microstructures^21^, such as shown in Fig. 1c, where a film of fluorescein has evolved nanolobes of 500 nm diameter. The effect of source temperature and deposition duration on size and shape of the structures has been previously demonsrated^20, 21^. Unlike other vapor-based deposition techniques (VTE, chemical vapor deposition, atomic layer deposition, and others), OVJP can be performed at atmospheric pressure and in some cases in ambient atmosphere^24, 25^. These characteristics and the fact that the OVJP apparatus itself is very simple suggest that OVJP is a perfect candidate for the multiple uses outlined above.

OVJP^23–25^ is unlike traditional ink-jet printing in that it operates without liquid solvents, eschewing the need to pre-dissolve the API^26, 27^, in principle making it attractive for application to printing APIs. However, typical API films deposited onto 1 × 1 cm^2^ surface area should in some cases be thicker than the organic semiconductor films used in optoelectronic devices, e.g., on the order of 10–1000 nm vs. 10–100 nm. How does the (potentially prolonged) evaporation process affect the chemistry, structure, and functionality of a drug compound? Will advantageous film and particle morphologies be obtained when the API is printed at useful thicknesses and onto biocompatible substrates? Here we address these questions and demonstrate how films of small molecular medicines can be made controllably by OVJP, obtaining simultaneously: crystalline deposits, enhanced dissolution kinetics and bioavailability, and precise dosages.

## Results

### Chemical and structural characterization

**Fig. 2:**
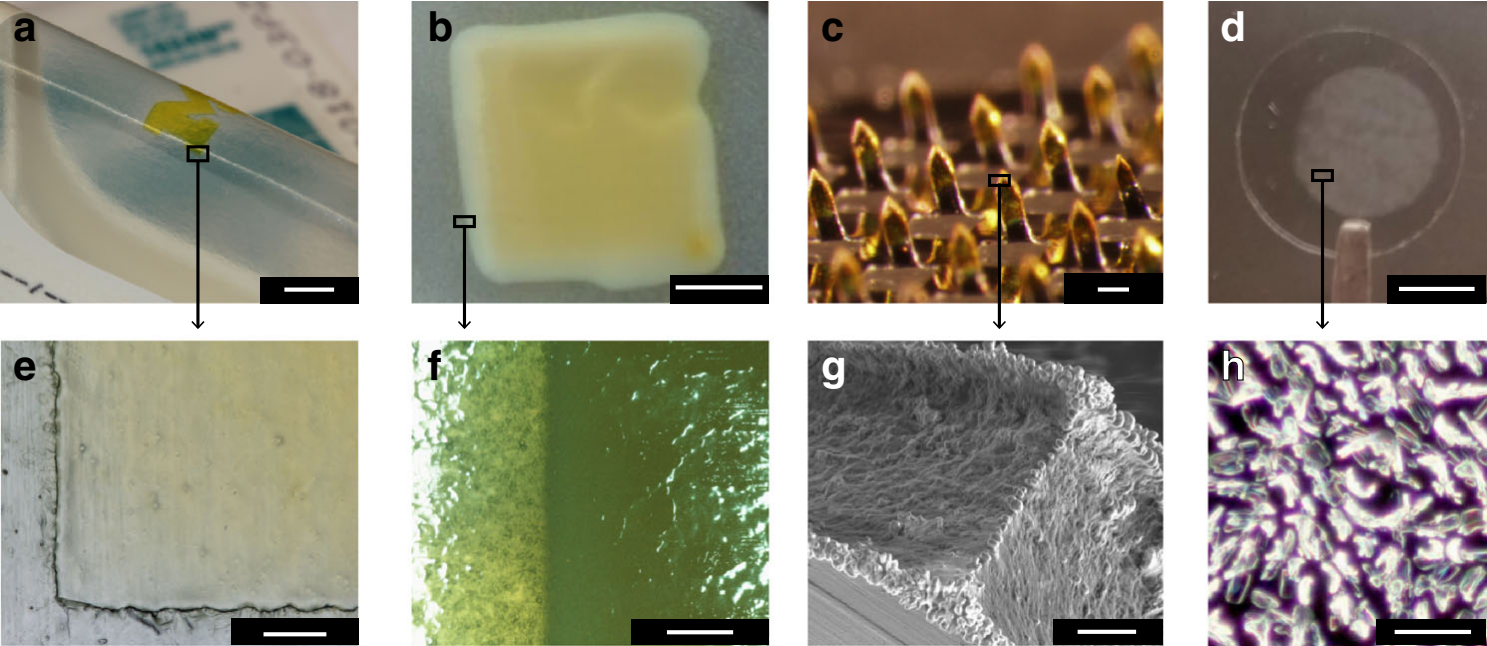
Examples of OVJP coatings on different, biomedically important substrates. **a**, **e** Fluorescein on 3M Tegaderm. **a** *Scale bar* 10 mm, **e** *scale bar* 500 μm. **b**, **f** Fluorescein on Listerine. **b** *Scale bar* 10 mm, **f** *scale bar* 500 μm. **c**, **g** Fluorescein printed on tips of stainless steel microneedles. **c** *Scale bar* 200 μm, **g** *scale bar* 10 μm. **d**, **h** Tamoxifen printed with OVJP onto borosilicate glass slides. **d** *Scale bar* 5 mm, **h** *scale bar* 20 μm

Figure 2 shows examples of vapor jet coating onto medical grade substrates: fluorescein patterned onto a 3M Tegaderm patch and a Listerine tab (Fig. 2a, b), fluorescein deposited onto a microneedle patch^28, 29^ (Fig. 2c), and tamoxifen deposited onto borosilicate glass slide (Fig. 2d). In all samples studied here, the film adheres to the substrate, and all tests are performed with the film adhering to the substrate.

Six different drugs—caffeine, paracetamol, ibuprofen, tamoxifen, fluorescein, and BAY 11-7082 (BAY)—were characterized by thermogravimetric analysis (TGA)^30^ to determine, establishing the conditions for deposition (i.e., flow rate of carrier gas, source temperature, and substrate temperature, as summarized in Table 1). Then, 9 mm diameter circular films were deposited onto borosilicate glass slides (Fig. 2d) using identical nozzle geometry in each case, with the deposits characterized using x-ray diffraction (XRD), chemical analysis, and high-resolution optical and electron microscopies.

**Table 1:** OVJP processing conditions for materials used in the study. Source temperature was determined via thermogravimetry and tuned to obtain a local deposition rate of ~0.5 μg per min

**Process parameter/ Material**	**Carrier gas type**	**Carrier gas rate (sccm)**	**Source temperature (** ^**°**^ **C)**	**Substrate temperature (** ^**°**^ **C)**
Fluorescein	Nitrogen	200	300	20
Caffeine	Nitrogen	100	130	20
Tamoxifen	Nitrogen	100	115	20
BAY 11-7082	Nitrogen	100	90	20
Paracetamol	Nitrogen	100	190	20
Ibuprofen	Nitrogen	150	75	20

**Fig. 3:**
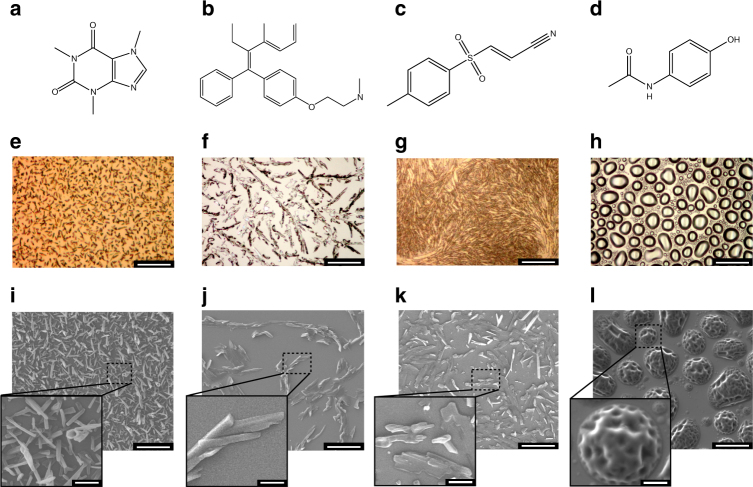
Chemical formulae of compounds used in this study, along with optical (*color*) and electron (*gray-scale*) micrographs of resulting films. **(a, e, i)** Caffeine, **(b, f, j)** Tamoxifen, **(c, g, k)** BAY 11-7082, **(d, h, l)** Paracetamol. The *scale bar* in **(i, j, k, l)** is 10 μm, with the *inset scale bar* 2 μm

Examples of the obtained film microstructures are shown in Fig. 3. All films were evaporated from original coarse powders whose particle sizes ranged between 1–100 μm (Supplementary Fig. 1). The caffeine coating consisted of needle-like features whose diameter and length were 400 ± 100 nm and 3 ± 1 μm, respectively (Fig. 3e,i). Tamoxifen films consisted of continuous platelet-like features 800 ± 100 nm and 500 ± 100 nm in height and width, respectively (Fig. 3f,j). BAY 11–7082 films also consist of discrete platelets-like features 800 ± 100 nm and 500 ± 100 nm in height and width, respectively, and 5 ± 2 μm in length (Fig. 3g,k). With paracetamol (Fig. 3d,i) and ibuprofen (Supplementary Fig. 2), 10 μm diameter, crystallized, interconnected droplets were obtained. Particle size estimation is described is Supplementary Fig. 3.

We attribute the latter morphology to the fact that paracetamol and ibuprofen were deposited at temperatures of 190 and 75 °C, respectively, which were 20 and 5 °C above their melting point, suggesting that adsorption took place via a transient liquid phase, followed by dewetting and crystallization. In this case, the deposit morphology can be tuned by changing the source temperature, carrier gas flow rate, and adjustments to the deposition flux.

**Fig. 4:**
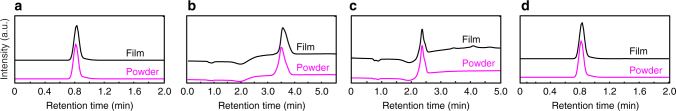
Drug films and powders chemical characterization. UPLC of: (**a**) caffeine, (**b**) tamoxifen, (**c**) BAY 11-7082, (**d**) paracetamol

To test the chemical stability of deposited substances, drug films were dissolved in methanol and analyzed by ultra-performance liquid chromatography (UPLC), for comparison to as-received API powder. Similar retention times and peak structures were obtained for deposited films and the original powder samples, (Fig. 4a–d) suggesting that the OVJP process did not alter the chemical structure of the API. Additional tests of BAY 11-7082 film with FTIR (Supplementary Fig. 4) also revealed no chemical degradation of the material.

**Fig. 5:**
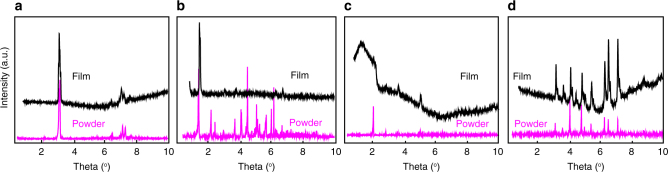
Structural characterization of drug powders and deposited films. X-ray diffraction patterns of: (**a**) caffeine, (**b**) tamoxifen, (**c**) BAY 11-7082, (**d**) paracetamol

Note that for pharmaceutical applications, polymorphism can change drug effectiveness and functionality^31, 32^. Hence, the crystal structure of the API films was studied using synchrotron X-ray diffraction and compared to the structure of the original powder and reference experimental data. The diffraction patterns of films were in very good agreement with both bulk powder used in the evaporation source and reference data available on stable materials polymorphs, indicating that OVJP did not change the crystal structure/polymorph composition of the API (Fig. 5a–d, Supplementary Figs. 5–9). The obtained crystal phase in printed caffeine film was β-form caffeine^33–36^, and form I in case of paracetamol^37–39^. In the case of tamoxifen base, its crystal structure was similar to that of the powder and to the only phase reported in literature^40^. The broadening of diffraction peaks in the printed films is attributed to reductions in the average size of the crystallites^41^, compared to that in the original powder. This reduction in crystallite size upon printing occurs due to supersaturation of the API vapor above the substrate, which reduces the critical nucleus size in the formation of new crystallites throughout the deposition process, discussed elsewhere^21, 25^.

### Dissolution testing

We now compare the dissolution kinetics of APIs in powder form to that of films printed by OVJP. Accepted pharmacokinetic equations will be used to fit the dissolution data, and key differences will be highlighted, showing that for drugs printed as films, common limitations of powder dissolution do not exist. This outcome is then used to demonstrate the dosing of poorly soluble drugs in their pure form in a biological system.

To characterize how crystallite size affects the dissolution process, the Noyes–Whitney^9^ equation is often used:

1 $$\frac{{{\rm{d}}C\left( t \right)}}{{{\rm{dt}}}} = \frac{{DA\left( t \right)}}{{V\delta \left( t \right)}}\left( {{C_{\rm{s}}} - C\left( t \right)} \right)$$

where *C(t)* is solute concentration as a function of time, *t* is time, *D* is diffusion coefficient in the solvent, *A* is solute-liquid contact area, *V* is solvent volume, *δ* is boundary layer thickness, *C*_s_ is the solubility limit in a given solvent. While *A* can sometimes be measured, boundary layer behavior is often a confounding and poorly controlled variable. OVJP enables the small molecular API to be formed as a continuous film. Although the surface profile may exhibit nanoscale roughness, this fine-scale roughness helps to quickly establish a virtual surface—i.e., a saturated boundary layer, which remains at a relatively constant thickness throughout the dissolution process, and therefore opens a simplified way of controlling and studying the dissolution process of a drug. Hence, for OVJP-deposited API films, Eq. (1) simplifies to:

2 $$C\left( t \right) = {C_{\rm{s}}}\left( {1 - {\rm exp}^{ - \frac{{DAt}}{{V\delta }}}} \right)$$

For the sink condition (*C*<<*C*_s_), the dissolution rate becomes essentially constant, precisely controlled by the film’s projected area.

To characterize powder dissolution kinetics, the Hixson and Crowell model^42^ is a simplified solution to Eq. (1):

3 $$C\left( t \right) = \frac{N}{V}\left[ {{\rm{M}}{{\rm{p}}_0} - \left( {{\rm{M}}{{\rm{p}}_0}^{1/3} - {{\left( {{{\left( {\frac{{4\pi }}{{3{\rho ^2}}}} \right)}^{1/3}}\frac{{D{C_{\rm{s}}}}}{{3\delta }}} \right)}^{\!\!\!3}}t} \right)} \right]$$

where *N* is the number of powder particles, Mp_0_ is the init﻿ial weight of the particles comprising the solute, and *ρ* is solute density. The active contact area in powder dissolution is, in fact, changing during the process, affected by changing particle shape, wettability, and tendency to agglomerate—effects that the H–C model does not include^43^. Note that powder micronization techniques, used to increase the dissolution rate, usually suffer from powder agglomeration, where the effective *N*/*V* ratio is reduced, while the effective boundary layer thickness *δ* often is a strong function of time.

The dissolution kinetics of three materials having poor aqueous solubility were studied: fluorescein in deionized water^44^, ibuprofen in aqueous hydrochloride (HCl) buffer (pH = 1.2) solution^45^, and tamoxifen in aqueous acetate buffer solution^46^ (pH = 4.9). (Caffeine exhibited instantaneous dissolution in film form, making it impractical to measure its dissolution kinetics in the film form. BAY 11-7082 was not tested due to low absorbance signal at tested concentrations.) First, the solubility of these compounds in corresponding solvents was measured at 20 ± 1 °C. The saturation limit for fluorescein in deionized water was measured to be 10 ± 0.5 μg ml^−1^, for ibuprofen in HCl 22.5 ± 0.5 μg ml^−1^, and for tamoxifen in acetate 23.6 ± 0.5 μg ml^−1^. To measure dissolution kinetics, a rotating disk dissolution apparatus (see Supplementary Fig. 10 for details) was used with rotational speed of 100 r.p.m. Concentration was monitored using an ultraviolet-visible (UV-VIS) spectrometer equipped with a dip probe. The compounds were tested both in powder form (compressed into pellets for rotating disk dissolution) and in film form. The intrinsic dissolution rate (IDR) of the films was compared to the dissolution rate of powder compressed into 1.57 mm diameter pellets of ~4 mm thickness^47^ (Supplementary Fig. 10b), while the printed films comprised 9 mm diameter circles on borosilicate glass disks (Supplementary Fig. 10c). Film weights were in the range 5–80 μg. Identical rotation rod and sample attachments were used (Supplementary Fig. 10a), that the hydrodynamic boundary layer thickness is same for compressed powder and deposited film. Solution volume remained constant in all experiments, 10 ml, and temperature was 20 ± 1 °C. The IDR is defined as:

4 $${\rm{IDR}} = \frac{{{{\left( {{\mathrm{d}m}/{\mathrm{d}t}} \right)}_{\rm max}}}}{A}$$

where *m* is the dissolved solute mass, and (dm*/*dt)_max_ is the maximum slope in the dissolution curve that plots the amount of dissolved material vs. time, typically evaluated at the start of dissolution process, where the concentration driving force is highest. IDR is an intrinsic property of a dissolving material, which depends for a given solvent on the material’s degree of crystallinity, crystalline and chemical structures. In all cases, intrinsic dissolution of films was comparable to one of compressed pellets (3·10^−5^ ± 5·10^−6^ for fluorescein, 1·10^−3^ ± 3·10^−4^ for ibuprofen, 6·10^−4^ ± 1·10^−4^ for tamoxifen, all values in (μg^s−1^ mm^−2^))). In the case of printed films, IDR was essentially unaltered, indicating that the material's chemical and structural form was not altered either, corroborating XRD and UPLC results.

**Fig. 6:**
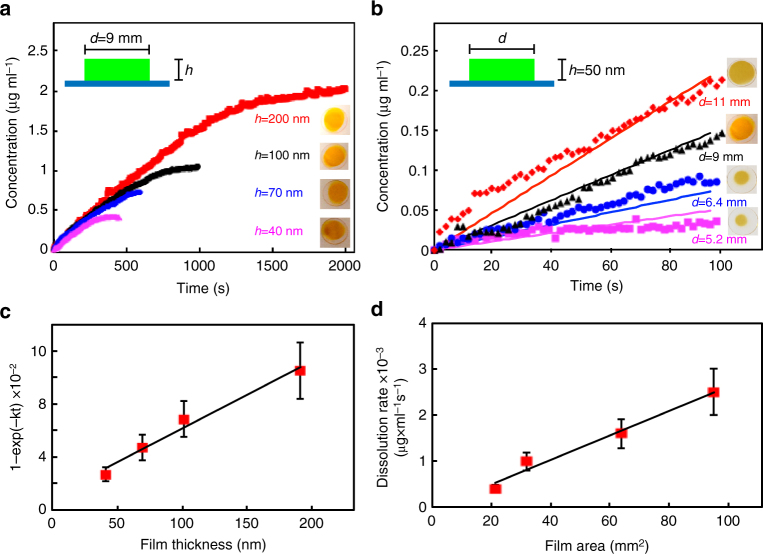
Example of controlled dissolution in fluorescein films. Effect of varying film thickness (**a**) or film area (**b**) on dissolution profiles of fluorescein films in deionized water. **c** Dependence of (1−e^*−kt*^) (Eq. 2) on film thickness. Numbers were extracted from data in plot **a**. **d** Films dissolution rate vs. film area. The rates were extracted from data in plot **b**. Error bars correspond to measurement of at least three samples ± standard deviation

Figure 6 demonstrates how the dissolution rate of films can be controlled via film thickness and deposit area. For example, keeping film area constant, while varying the thickness of the fluorescein film (Fig. 6a), we observe identical IDRs, while the final concentration of fully dissolved sample increases linearly with film thickness; this is confirmed by plotting the right hand side of Eq. (2) in Fig. 6c. Figure 6b shows how concentration varies with deposit area, while keeping the thickness constant. The concentration evolves essentially linearly with time, with the slope proportional to the deposit area, as indicated by a linear fit in Fig. 6d, from which the IDR can be extracted. The dissolution rate scales linearly with film area, as predicted by Eq. (3).

**Fig. 7:**
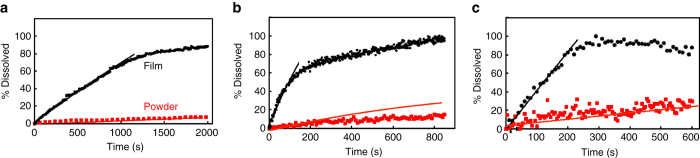
Dissolution profiles of films and powders. Dissolution profiles of films and original powders (*dotted line*—experimental values. *Solid lines*—theoretical prediction according to Eq. 2 for film and Eq. 3 for powder): (**a**) fluorescein in deionized water—23 μg weight. **b** Ibuprofen in aqueous HCl buffer pH 1.2—70 μg weight. **c** Tamoxifen acetate buffer pH 4.9—5.5 μg weight

As-received API powders having the same weight as the films were introduced into 10 ml solutions without any prior treatment, and stirred using a rotation rod with the same shape and diameter as the one used for film dissolution. Figures 7a–c demonstrate the dissolution behavior of films vs. original loose powders, clearly showing that the initial dissolution rates in films is very rapid and constant up to ~80% of the film being dissolved. The dissolution rate is diminished for the remaining material mainly due to reduction in film area (i.e., loss of complete coverage due to material that has dissolved). The initial dissolution rates observed for film-form materials vs. loose powders are enhanced 10-fold for fluorescein, 30-fold for ibuprofen, and 10-fold for tamoxifen. As hypothesized, the initial enhancement in dissolution rate is attributed mainly to the enhancement of surface area of the film, while the IDR ultimate solubility remain constant. The order of enhancement is in good agreement with the order of enhancement of surface area relative to that of powders. Importantly, film dissolution accurately follows predictions until almost complete dissolution, whereas in the case of powders, the dissolution rate is less predictable due to changes in particle shape and agglomeration^35^ over time, clearly present, for example, in the dissolution of ibuprofen powder (Fig. 7b). Ιt is likely that the very high surface area of the vapor-deposited films leads to relatively fast establishment of an equilibrium-limited concentration in the boundary layer above the surface, maximizing the driving force for dissolution.

### Biological testing

To determine in a controlled manner whether enhanced API dissolution rates translate effectively to the API’s action on live cells, two different cancer cell lines (ovarian carcinoma, OVCAR3, and breast carcinoma, MCF7) in growth medium were exposed to tamoxifen films and BAY 11-7082 films printed on glass slides. The cells were plated onto 12-well tissue culture dishes, and allowed to adhere and grow for 24 h in 2.5 ml of 10% fetal bovine serum-supplemented growth medium. Tamoxifen and films were inverted and placed onto the surface of the growth media (Supplementary Fig. 11), and released from the substrate via gentle, intermittent manual agitation over the course of 1 h. Slides were removed following this initial period, and live cell counts were determined using Trypan blue exclusion using a hemocytometer at 1, 4, 8, 12, 24, and 48 h following film treatment (Supplementary Fig. 12). At least three samples were tested at each time point. Growth inhibition curves were also generated using the following controls: clean glass slides with no deposited drug film as a sham control; 5 µM tamoxifen or 500 nM dissolved in DMSO (conventional drug dose); and tamoxifen or BAY powders dissolved directly in sterile supplemented growth medium. In all cases, the amount of the introduced drug was calculated such that the nominal concentration of the treatment was 5 µM (1.8 µg ml^−1^) for tamoxifen (4.5 µg per film) and 500 nM (0.1 µg ml^−1^) for BAY 11-7082 (0.25 µg per film).

**Fig. 8:**
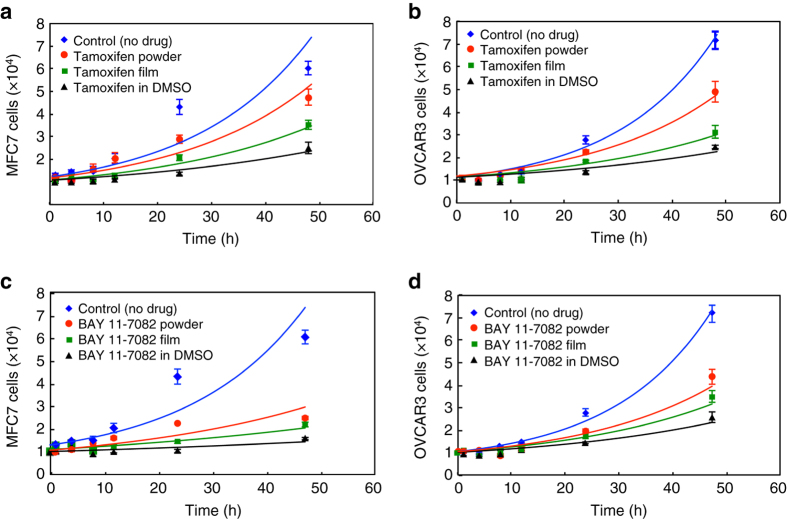
Cell growth studies. Cell growth profile (*solid line*—eye guide): **a** MCF7 cells treated tamoxifen, (**b**) OVCAR3 cells treated with tamoxifen, (**c**) MCF7 cells treated with BAY 11-7082, (**d**) OVCAR3 cells treated with BAY 11-7082. All data points in the plots are an average of at least three film samples ± standard deviation

Figure 8 demonstrates cancer cell count curves treated with the different drug forms. All data points in the plots are an average of at least three film samples. In both cases, cells treated with film-form drug showed significantly reduced viability, comparable to when the drug was pre-dissolved in DMSO. In case of treatment with tamoxifen, MCF7 cancer cells’ viability after 48 h was 58% for film form and 79% for powder-form treatment (Fig. 8a), and OVCAR3 cancer cells’ viability after 48 h was 44% for film form and 68% for powder-form treatment (Fig. 8b). Films exhibited similar effectiveness as the powdered drug dissolved in growth medium (Fig. 8c, d). Importantly, DMSO alone and neat substrate alone did not show an impact on the cancer cell growth as shown in Supplementary Fig. 13. The greater observed effectiveness of film-form dosing of tamoxifen is attributed to faster dissolution and, hence, greater effective concentration of the drug relative to that obtained from powders.

## Discussion

In summary, we report on the solvent-free printing of small molecular pharmaceutical ingredients to obtain high-surface area films having nanocrystalline morphology. These printed films exhibit substantially enhanced dissolution kinetics compared with their bulk powder form. Drug films printed in this manner (shown here for tamoxifen) exhibit enhanced cancer cell-killing properties in vitro, without having to pre-dissolve them in organic solvents, such as DMSO. While amorphous dispersions have been used to increase the APIs’ solubility, their stability remains questionable^48^; the nano- and microcrystalline nature of the printed films suggests a promising avenue for breaking the trade-off between stability and dissolution rates.

More generally, the ability to deposit small molecular drugs from their pure form, as films and without the use of solvents as shown here, opens an alternative approach to drug screening and manufacturing, where accurate dosage, chemical and structural stability, and processing flexibility are needed without hindering drug functionality. We therefore anticipate benefits for: early-stage drug discovery—eliminating the need for organic solvents for poorly soluble drugs during screening processes, development of drug delivery vehicles—e.g., films for transdermal drug delivery, directly coated patches, microneedles, encapsulated dissolvable films or implants, manufacturing and administration of HPAPIs with accurate, individualized dosing and nanogram-level accuracy during manufacturing. The technique demonstrated here also potentially enables continuous manufacturing, ^23, 49^, eliminating the need for mixing and powder preparation.

## Methods

### Film fabrication

Caffeine (C0750) >99% was purchased from Sigma-Aldrich. Tamoxifen (ICN15673883) >99.24% and paracetamol (5074863) >99.5% were purchased from Fisher Scientific. BAY 11-7082 (19542-67-7) >98% and ibuprofen (401003) >99% were purchased from EMD Millipore, fluorescein (32615) >99.8% was purchased from Fluka; all were used as received. Borosilicate glass slides, 12 mm diameter, and 0.2 mm thick, were used as substrates for film deposition. Substrates were cleaned by ultrasonication in detergent solution and deionized water, followed by acetone and isopropanol rinses, for 10 min each. Substrates were then placed in boiling isopropanol for 5 min and dried in pure nitrogen gas prior to film deposition.

### OVJP process parameters

OVJP nozzles used in this study were made from quartz tubes having 12.5 mm outer diameter and a nozzle tip of 500 μm internal diameter, with an ~15° inside wall taper from the nozzle axis. The inert carrier gas was 99.99% pure nitrogen.

The nozzles were cleaned with acetone and isopropanol solvents, dried and wrapped with 36″ gauge heavy insulated tape heater (Omega Engineering, Inc.) with a power density of 8.6 W·in ^−2^. The heating tape leads were connected to a temperature controller (Digi-Sense Benchtop temperature controller, Cole Palmer Instruments Co.), and a 1/16 inch diameter K type thermocouple was used to monitor and maintain the temperature of the source. The source consisted of ~0.15 g of powder embedded in a porous SiC ceramic foam of 100 dots per inch (DPI) and placed in the heated source section of the tube. The gas flow rates were maintained using mass flow controllers (C100 MFC, Sierra Instruments).

The process parameters that were kept constant are: nozzle-substrate separation distance (1.5 mm) and substrate temperature (20 °C). The process was performed in glove box purged with 99.99% pure nitrogen gas. Detailed processing conditions are given in Table 1.

Deposits were formed by rastering the nozzle over the substrate to form adjacent, overlapping lines at 0.2 mm center-to-center spacing, allowing for homogeneous thickness of deposit for a nozzle of 0.5 mm inner diameter positioned 1.5 mm from substrate surface. Fluorescein films on microneedles were deposited through a flexible mask. The same process can be performed without a mask when using nozzle with appropriate printing resolution.

### X-ray measurements

Surface X-ray diffraction measurements of the films were performed at Sector 13-BM-C at the Advanced Photon Source, Argonne National Laboratory using a Newport 6-circle kappa diffractometer. Figure 5 scans were obtained by specular diffraction (2-theta scan) in which the incident angle was fixed at grazing incidence and the detector scanned the scattered 15 keV X-ray beam. This was done to increase the sampling volume because the films were polycrystalline. The diffracted intensity was collected using a Dectris Pilatus 100 K pixel array detector. All measurements were performed in a helium-rich environment to prevent rapid degradation of the organic films. Diffraction measurements of powders were performed with Rigaku rotating anode XRD using monochromatic Cu-K-alpha X-ray source. Differences in peak intensities between the powder and film measurements are due to variation in the setups used. The broad feature in Fig. 5c below 1.8° degree is a substrate artifact that could not be eliminated due to the very low angle nature of the measurement.

### Microscopy

An FEI Nova 200 Nanolab scanning electron microscope with accelerating voltage of 5–10 kV and current 0.1–0.5 nA was used to obtain the surface morphology images. Optical microscopy analysis was performed with Zeiss microscope (bright field, top-illumination).

### Thermogravimetry of pharmaceutical substances

To determine evaporation temperature of the powders, and subsequently source temperature in the system, TGA was used. All measurements were performed using a TA Instruments Thermogravimetric Analyzer Q500 at 0.01% accuracy, with nitrogen sample purge flow rate 60 ml per min and balance purge flow rate of 40 ml per min. Heating rate was 5 °C per min.

### UV–VIS measurements

Drug dissolution was monitored by in situ UV–VIS Ocean Optics USB + 2000 spectrometer with T300-UV–VIS transmission dip probe, with a 10 mm optical path. Solution absorbance was recorded with frequency of 2–60 s^−1^ for duration of 20 min–12 h (depending on substance dissolution rate, fast dissolution rate was recorded at higher frequencies). *λ*_max_ for absorbance testing was: 490 nm for fluorescein, 224 nm for ibuprophen, and 240 nm for tamoxifen.

### UPLC

To determine the concentration of analyte, and to screen for degradation or impurities, drug powders and films were dissolved in methanol and run on a Waters Acquity H Class UPLC equipped with a Waters Acquity UPLC C18 column (2.1 × 100 mm). Drug powders were used as standards in all cases, and were diluted such that film samples fell within the range of standards. For all molecules, the mobile phase was pumped at 0.3 ml per min, and consisted of A: ddH_2_O + 0.1% formic acid and B: methanol + 0.1% formic acid. The detection wavelength was set to 280 nm. Caffeine and paracetemol were run isocratic at 100% B, while Tamoxifen and BAY had a gradient elution starting at A/B: 75/25, to 25/75 at 0.5 min, steady through 5 min, then returned to 75/25.

### Dissolution rate measurement

For dissolution of the film, the glass substrate with the printed film was attached to a rotating rod with a substrate holder. In the case of compressed powder pellet, a 1.57 mm diameter pellet was compressed at 50 ± 2 psi for ~10 s into a holder having same size as disk substrate holder. In the case of loose powder, the weighed powder was dissolved into solution and stirred with blank substrate holder at 100 r.p.m. The detailed dissolution setup is shown in Supplementary Figure 10. In all cases, the solution volume was 10 ml, and vessels were identical. Testing temperature was 20 ± 1 °C. Sink conditions were defined as C < 5Cs. Except for ibuprofen testing, the rest of experiments met sink conditions requirement.

### Solubility tests

Solubility tests were performed at ambient temperature (20 ± 1 °C). Solutions with excess concentration (at least 100 μg ml^−1^ of solid) were shaken at ~1000 r.p.m. for 48 h, followed by solution filtration with a 0.4 μm filter after 24 h. Filtered solution concentrations were then measured using UV–VIS spectrometry, calibrated with known solution concentrations. Five different concentrations for calibration were used in each test. The concentrations used were as follows: for fluorescein in dionized water −1, 2, 3, 4, 5 μg ml^−1^, ibuprofen in HCl pH 1.2 buffer: 2, 4, 6, 8, 10 μg ml^−1^, tamoxifen in acetate buffer pH 4.9: 1, 2, 4, 6, 10 μg ml^−1^.

### Theoretical modeling

Theoretical predictions were calculated according to Noyes–Whitney theory (Eqs. 2 and 3 in main text). The boundary layer thickness according to Levich theory^50^ was estimated to be ~40 μm. Since the boundary layer thickness in the rotating disk setup does not depend on compacted powder diameter, D was extracted for each compound based on compacted powder measurement and used for theoretical prediction of loose powders and printed films in plots for Figs. 6 and 7. The boundary layer thickness for powder particles was estimated to be the same as the particles' average size.

### Data availability

The data that support the findings of this study are available from the corresponding author on request.

## Electronic supplementary material

Supplementary Information
