# Supplementary material for: Printing of small molecular medicines from the vapor phase
Source: Nat Commun. 2017 Sep 27;8:711. doi: 10.1038/s41467-017-00763-6 (PMC5617892; doi:10.1038/s41467-017-00763-6)
Supplement: Supplementary file 1 — Supplementary Information [file 41467_2017_763_MOESM1_ESM.pdf]

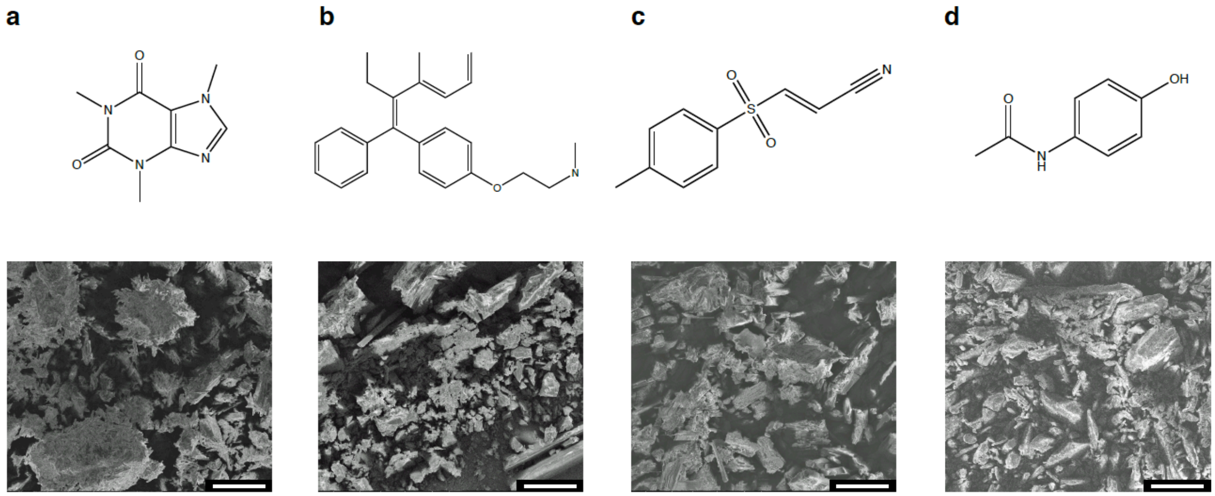

Supplementary Figure 1. Microstructure of original powders
(**a**) Caffeine, (**b**) Tamoxifen, (**c**) BAY 11-7082, (**d**) Paracetamol. Scale bar 50 μm.

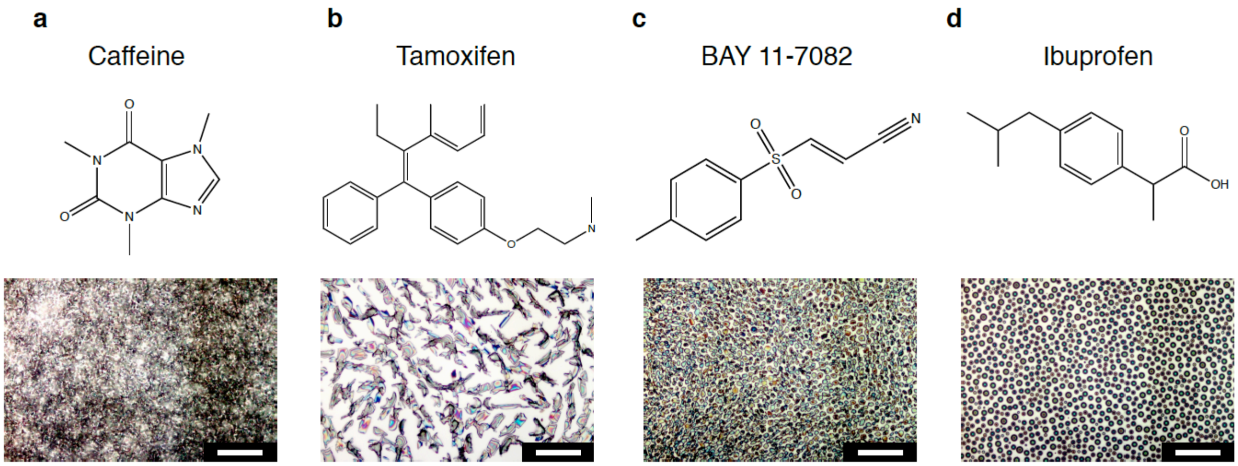

Supplementary Figure 2. Additional optical images of different drug substances printed on silicon (100). The resulting morphology depends on thermophysical properties of drug, substrate material and deposition conditions. Films were deposited at conditions described in **Table 1.** (**a**) Caffeine, (**b**) Tamoxifen, (**c**) BAY 11-7082, (**d**) Ibuprofen. Scale bar 20 µm.

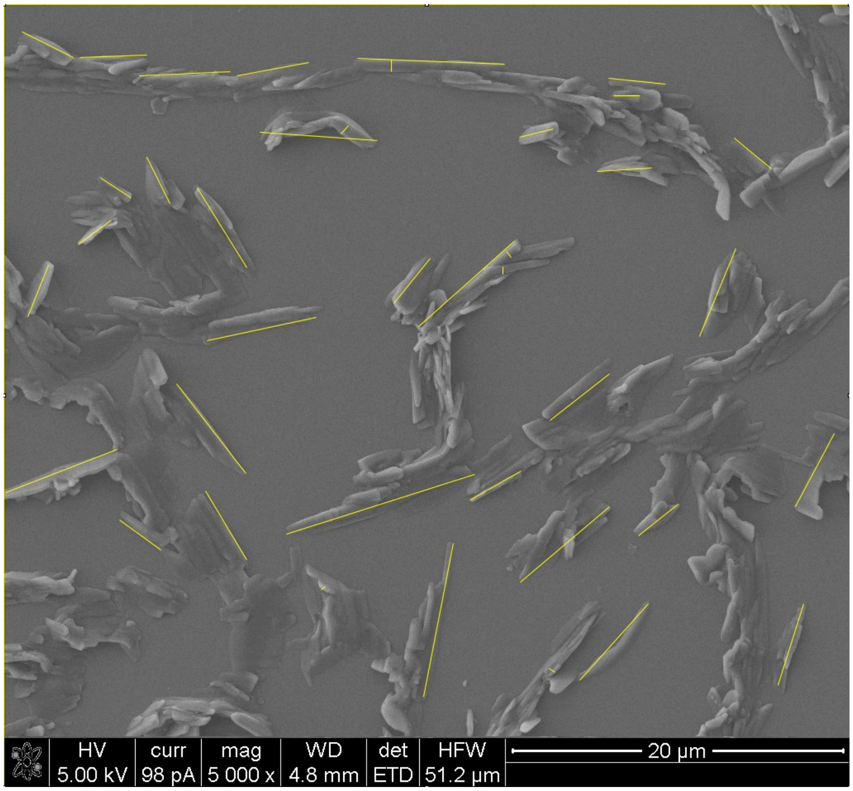

Supplementary Figure 3. Evaluation of particle sizes and surface area in powders and films

Powder and particles average sizes, shapes and surface areas were evaluated using Image-J software. In each case an average of at least 30 particles was measured. Based on the particles' shape and size, their average volume and weight were calculated. This micrograph is an example of measurement of length of tamoxifen film platelets grown on glass (yellow lines are calibrated with image scale bar). In a similar manner, tamoxifen platelets' width and height were measured, and volume and weight of rectangular prisms were calculated.

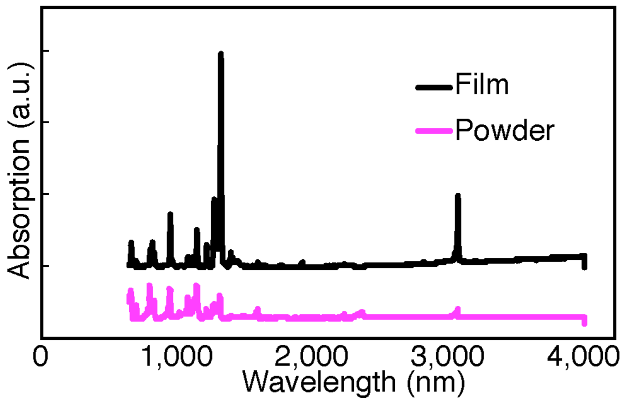

Supplementary Figure 4. FTIR absorption spectra of BAY 11-7082.

Absorption spectra of BAY 11-7082 film and original powder showed similar peaks, indicating that no material degradation occurred due to deposition.

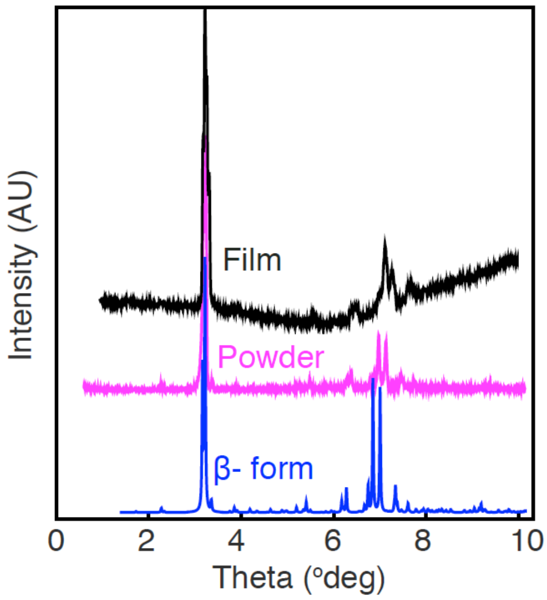

Supplementary Figure 5. Caffeine original powder and film form compared to β-form anhydrous caffeine

For caffeine, two polymorphs are usually reported: anhydrous β-form of caffeine that typically exists at low temperature, and high temperature α-form caffeine that exist above 141°C.

We compared XRD pattern of caffeine films and original powder to existing phases and found that both original powder and resulting film are in very good agreement with β-form of caffeine structure

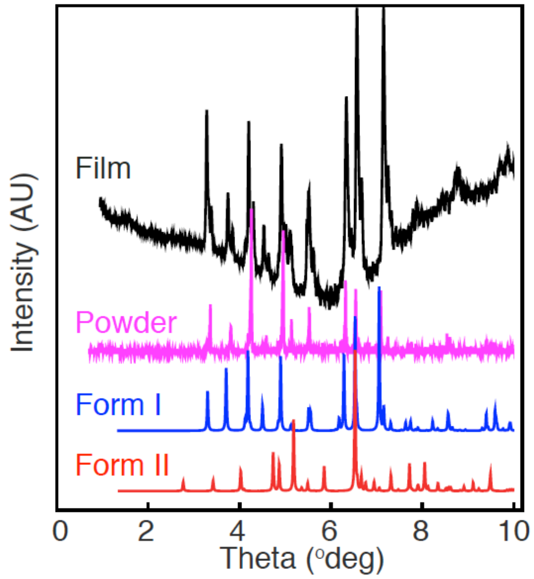

Supplementary Figure 6. Paracetamol original powder and film form compared to Form I and Form II paracetamol

Paracetamol was reported to have three common polymorphs, two of which were fully characterized: monoclinic Form I, most stable; orthorhombic Form II, less stable than I, and highly metastable Form III which is not fully characterized. Good agreement was found between the our samples and Form I paracetamol structure, both in powder and film samples, with a small amount of Form II admixed.

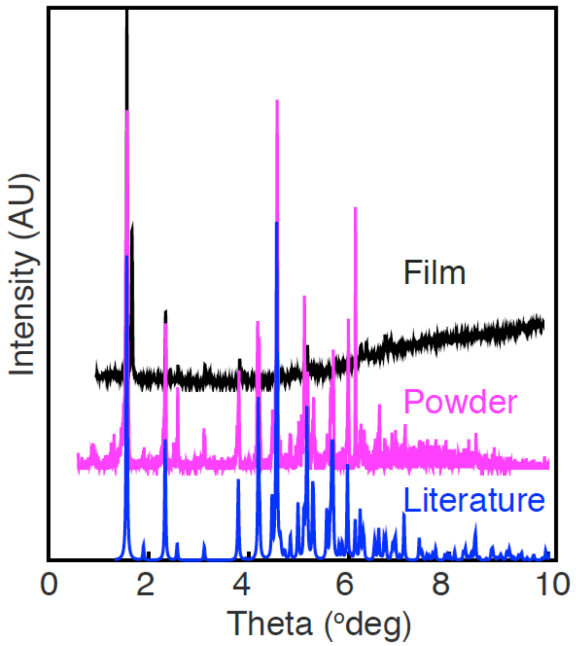

Supplementary Figure 7. Xray diffraction patterns of tamoxifen, original powder and resulting film, compared to literature data.

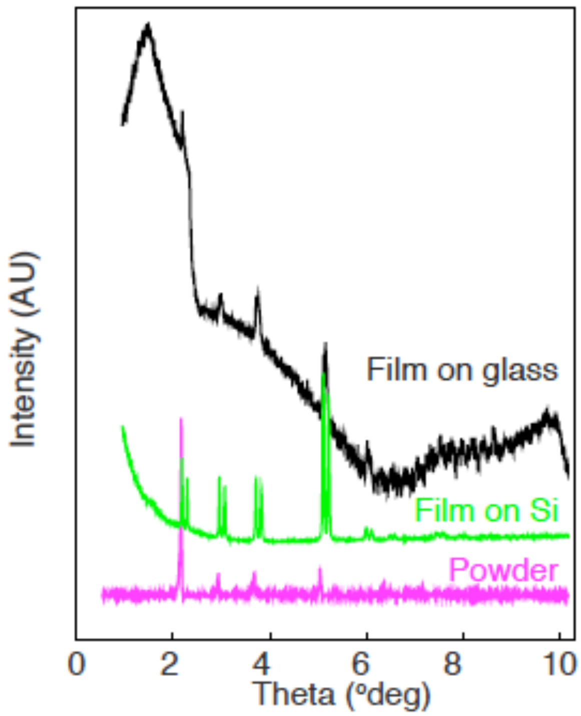

Supplementary Figure 8. Detailed diffraction pattern of BAY 11-7082 original powder and resulting films on glass and silicon substrates

In the case of BAY 11-7082, we found no previous reports of XRD data in the literature. The peak appearing below 1.8° is a measurement artifact, absent for example from the pattern from a film on a silicon substrate, also shown, which lacks this broad artifact.

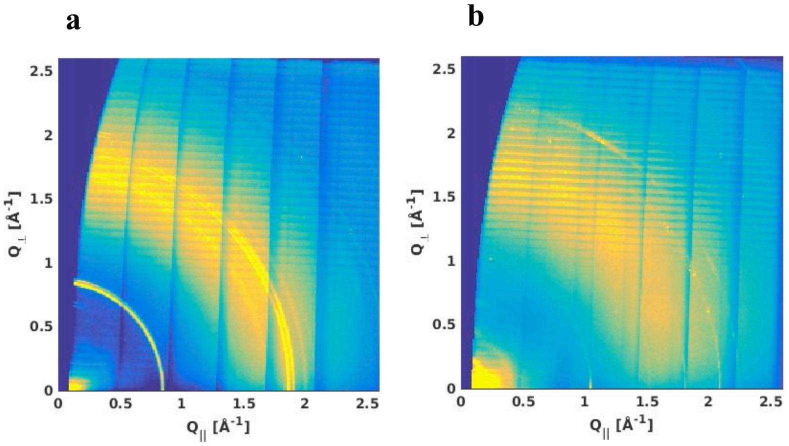

Supplementary Figure 9. Additional results of caffeine and ibuprofen films X-RAY diffraction

Additional results of films X-RAY diffraction. A monochromatic X-ray beam at 15 keV was used to probe the film's 3D crystal structure with the beam spot size optimized at 300 μm x 35 μm in the horizontal and vertical directions, respectively. To resolve the symmetry of the organic films, high-resolution Grazing-Incidence Wide-Angle X-ray Scattering (GIWAXS) measurements were performed. Using a PILATUS 100K area detector[51,52], the intensity distribution was measured in a series of detector scans along the out-of-plane direction as a set of two-dimensional reciprocal space slices. These were then used to reconstruct the 3D reciprocal space volume[53] from which 2D projections (reciprocal space maps) at given L values were derived as shown in the figures above. For each sample, the GIWAXS patterns were recorded at an angle of incidence of 0.1° and taken over a 2! range of 5° to 45°.GIWAXS patterns of (**a**) caffeine, and (**b**) ibuprofen films deposited on glass substrates. All films show nearly isotropic orientation with extremely broad and nearly uniform distribution of intensity for all rings in plane and out of plane. The broad rings suggest that caffeine and ibuprofen have a polycrystalline crystal structure when deposited on glass substrates. Qualitatively, caffeine films show the sharper rings than ibuprofen and are therefore more ordered.

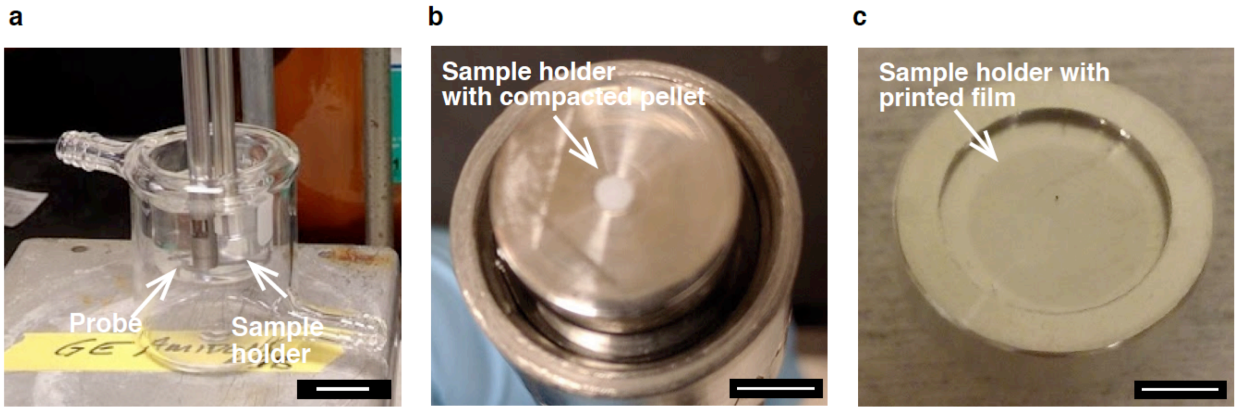

Supplementary Figure 10. Experimental setup used in dissolution rate studies

(**a**) Dissolution vessel with optical probe and sample holder. The rod is rotating at 100 rpm speed. (**b**) Sample holder for miniaturized compressed powder pellet (**c**) Sample holder for circular substrate with printed film. Scale bar 5 mm.

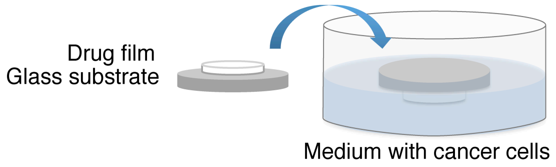

Supplementary Figure 11. Schematic of drug application for cancer cell growth study

Films printed on glass substrates were immersed into cell growth medium. After 1 hour of agitation, the glass film was removed.

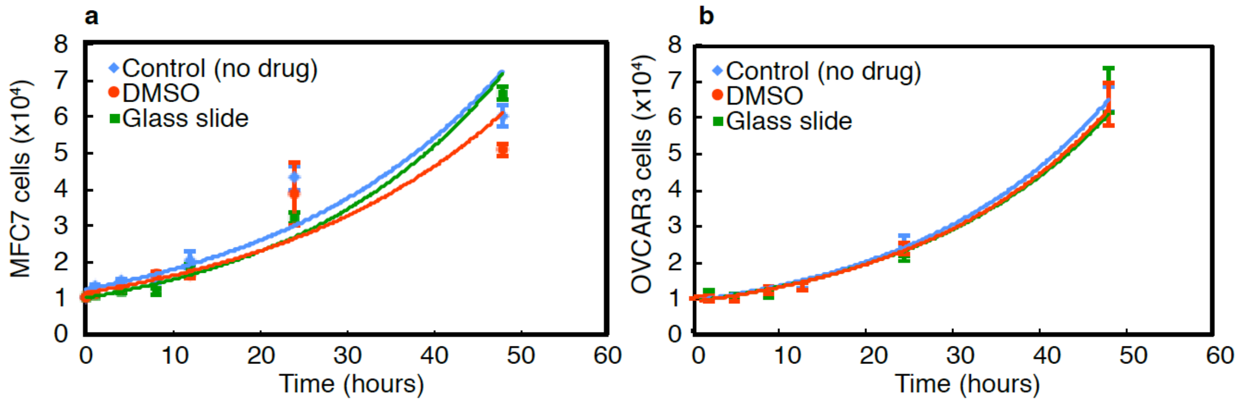

Supplementary Figure 12. Cancer cell growth curves – cell growth at drug-free conditions.

To ensure that glass slide doesn't affect cell growth, three drug-free control studies were performed: medium with just cancer cells, medium with just cancer cells and DMSO, medium with just cancer cells and glass slide. No effect of the presence of the bare glass slide on cell growth was observed in the case of breast cancer MCF7 (**a**) or ovarian cancer OVCAR (**b**) cell cultures.

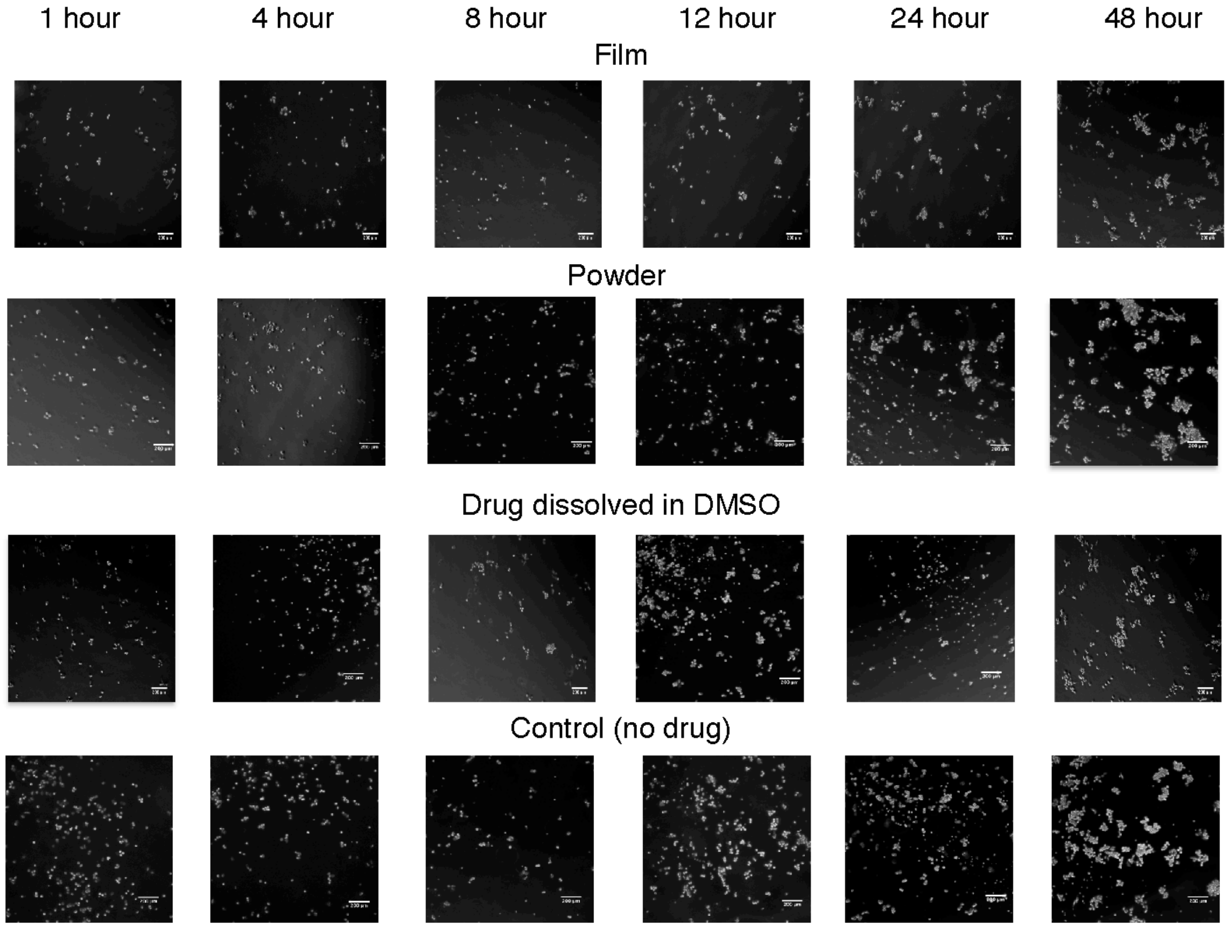

Supplementary Figure 13. Example of micrographs used for cancer cell population counts used in determining the growth curves.

Micrographs of MCF7 cancer cells treated with different forms of tamoxifen in media, used for cell counting.
